# Microbiota in the Early Lives of Sheep: A Short Overview on the Rumen Microbiota

**DOI:** 10.3390/ani16010080

**Published:** 2025-12-27

**Authors:** Antonio Bevilacqua, Suleman Khan, Mariangela Caroprese, Barbara Speranza, Angela Racioppo, Marzia Albenzio

**Affiliations:** Department of Agriculture, Food, Natural Resources and Engineering (DAFNE), Università of Foggia, 71122 Foggia, Italy; suleman.khan@unifg.it (S.K.); mariangela.caroprese@unifg.it (M.C.); barbara.speranza@unifg.it (B.S.); angela.racioppo@unifg.it (A.R.); marzia.albenzio@unifg.it (M.A.)

**Keywords:** sheep, prebiotics, probiotics, synbiotics, livestock, rumen, microbiota

## Abstract

A key area of research is the role played by the gut microbiota in the health of sheep during the early period of life; consequently, understanding its complexity may have significant implications for sheep farming. While factors such as diet, age, health status, and environmental conditions are crucial for determining the composition of the gut microbiota, there are very few summaries on the gut microbiota in early life and only a limited number of reviews considering the gut microbiota in adult (sheep) life. Accordingly, after a brief introduction to the generic qualitative and quantitative composition of the gut microbiota in sheep, this review focuses on its development in early life, with primary attention given to the rumen. It then provides an overview of factors that may modulate the microbiota, with a particular focus on prebiotics, probiotics, and synbiotics. The optimisation of these processes in sheep may enhance digestibility, immunity, and overall production efficiency. The review also highlights key challenges associated with the adoption of management strategies based on the integration of microbial approaches in modern livestock systems with the aim of enhancing animal welfare, sustainability, and production efficiency.

## Simple Summary

Sheep have millions of beneficial microorganisms in their guts, which play key roles in digestion, growth, and overall health. From the first weeks of life, microorganisms progressively colonise the various stomach compartments, particularly the rumen, which later becomes the primary fermentation chamber in young ruminants. In this context, this review presents an overview of how gut microbiota become colonised in young lambs, as well as the environmental, dietary, and direct-contact factors that influence colonisation. Furthermore, this review also explains the use of widely recognised natural additives, including prebiotics, probiotics, and synbiotics, which may improve the digestive system and disease resistance, as well as promote lamb growth in agricultural systems. This understanding of how the gut microbiota is assembled and organised during an animal’s early life may assist farmers and scientists in developing improved feeding strategies, thereby helping to prevent digestive disorders, and enhancing animal welfare.

## 1. Introduction: Composition of Sheep Gut Microbiota

The ruminant gastrointestinal tract (GIT) has a complex gut microbiome that maintains intestinal homeostasis, develops mucosal and lymphoid structures, and activates the host immune cell repertoire [1]. The gut microbiota of ruminants, as in many other animals, refers to the community of microorganisms residing in their digestive tract [2]. It plays a crucial role in their health, digestive functions as well as immunological properties such as interferon response [3]. Consequently, dysbiosis may lead to digestive disorders, reduced nutrient absorption and increased susceptibility to disease [4].

The species composition and diversity of the gut microbiota are influenced by various factors, including dietary manipulation, stress, antibiotic treatment, and the environment [5]. Therefore, research focused on the gut microbiota at early stages of development plays a crucial role in animal welfare, improved digestive efficiency, and animal productivity. A better understanding of these microbial interactions may also contribute to minimising the environmental impacts observed in agriculture, particularly those associated with methane gas formation in ruminant production [6].

**Figure 1:**
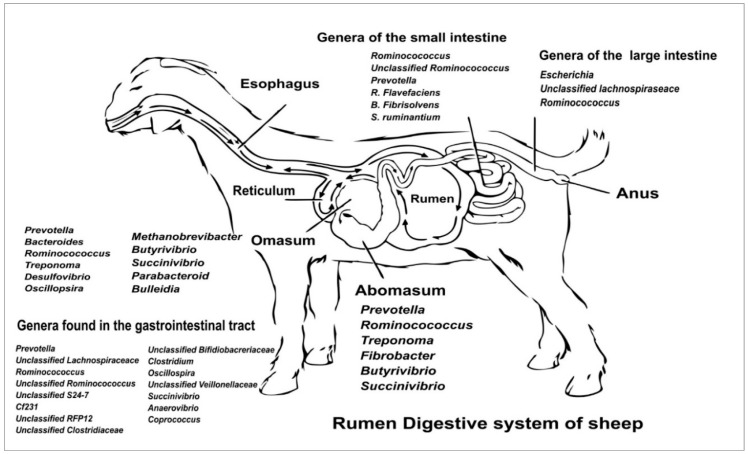
Microbiota composition in sheep gut.

The gut microbiota is composed of various microorganisms including bacteria, protozoa, fungi, and Archaea, all working together in a symbiotic relationship with the host to aid in the digestion of plant material and maintain overall gut health [7]. Differences in the average bacterial abundance at the family level found in the parts of the gastrointestinal system of the sheep are shown in Figure 1, as the microbiota composition is known to vary according to the tract (omasum, abomasum, and small or large intestine). *Prevotella* has been reported as one of the most important genera in the GI of ruminants [8], and is identified mainly in the rumen [9]; additionally, *Ruminococcus* spp. are reported as a part of the gut microbiota, including the rumen, with a predominance at the species level of *R. flavefaciens* [10]. Finally, *Bacteroides*, *Ruminococcus*, *Lactobacillus*, *Flavonifractor*, and *Clostridium* are the dominant genera in the caecum and rectum of Small-Tail Han sheep [11].

However, while many studies have examined the gut microbiota of adult sheep, few have specifically addressed early-life rumen colonisation. Accordingly, the main topic of this short review is an examination of the rumen microbiota in newborn and young lambs, as well as key important factors affecting its composition.

## 2. Methods

A literature search was conducted on Scopus and Web of Science, using the keywords rumen microbiota, early life, diet, sheep, lamb, probiotics, prebiotics, synbiotics, or combinations thereof. In the first screening step, articles published from 2010 onwards were selected; older articles were considered eligible only if they were the primary sources of essential information.

Consequently, after removing duplicates, a total of 190 articles were identified and progressed to the second screening step. At this step, screening was performed on abstracts, and only articles clearly focusing on the gut and rumen microbiota during the early life of sheep were retained. The final selection produced a total of 45 articles directly addressing the review topics, while an additional 50 articles were included due to their relevance to the broader thematic context.

## 3. Microbiota Colonisation and Development During Early Life

The rumen microbiota rapidly develops after birth and continues interacting with its host [12] as a result of multiple influencing factors, among which nutrition plays a crucial role [13]. Most ruminants’ GITs are assumed to be free of microbes at birth [14]; however, following delivery the rumen is rapidly colonised by bacteria within the first 28 days of life [15]. By contrast, Bi et al. [16] reported the presence of a microbiome with limited diversity and biomass in the foetal guts of lambs delivered by aseptic hysterectomy. Accordingly, some researchers have suggested that the womb may represent the starting point for the development of the maternal gut microbiome and for an initial microbial colonisation of the foetal gut [16].

Newborn lambs are similar to monogastric animals, and milk enters the abomasum via the oesophageal groove. Subsequently, rumen development occurs following a non-rumination phase, through a transition phase (3–8 weeks), and a rumination phase (from 8 weeks onwards) [15].

The rumen microbiota is composed of prokaryotic microorganisms, including bacteria and Archaea, and eukaryotic species such as fungi and protozoa [17]; these microorganisms act synergistically to support feed digestion and nutrient absorption [13]. Most studies have focused on bacteria, as they are essential for mature GIT function; however, the qualitative composition of the microbiota is strongly affected by age and may change in the space of a few days. A synopsis of the most important changes from birth to 40 days is reported in Table 1.

*Bacteroides*, followed by *Ruminococcus*, *Eubacterium*, *Prevotella*, *Bifidobacterium*, *Acidaminoccus*, *Clostridium*, *Veillonella*, and *Streptococcus* were predominant, accounting for 88.7% of the bacterial population in the calf rumen at birth [7,18]. Furthermore, exclusively milk-fed lambs exhibit a rumen bacterial microbiota characterised by early colonisers, such as *Ruminococcus albus*, *R. flavefaciens*, *R. ruminicola*, and *Eubacterium ruminantium*, which may degrade plant polysaccharides and promote rumen colonisation in sheep [19]. This observation is supported by other studies reporting that the first week of life is characterised by typical rumen bacteria, indicating cellulolytic bacteria colonisation even in the absence of solid nutrition [20].

In lambs, there are three successive phases in which rumen bacterial colonisation has been identified, similar to those described in calves; namely, 0–3 days, 10–20 days, and 20–56 days [21]. Similarly, Zhang et al. [22] demonstrated that three distinct phases characterise rumen microbial colonisation in goat kids: an early phase (0–14 days after birth), a transition phase (14–30 days), and a very steady phase (28–60 days). Early colonisers are essential for shaping immune tolerance and promoting pathogen resistance. As the lamb matures, the microbial community undergoes a transition stage, driven mainly by the consumption of fibre; the most affected taxa include *Prevotella*, *Bacteroidales*, *Ruminobacter* and *Selenomonas* (>5%), which show a greater abundance after 14 days [23]. During this transition phase, these mature taxa contribute significantly to lamb development by degrading plant fibres, facilitating nutrient acquisition, and further influencing the immune system, as previously demonstrated [24]. This dynamic shift in microbial composition underscores the importance of early microbial colonisation and its long-term impact on the lamb’s health and immune function.

Archaeal communities represent only 3–4% of the rumen microbiota; however, methanogenic archaea are phylogenetically distinct from other archaea due to their specialised role in methanogenesis [25]. This phylogenetic difference arises from unique metabolic pathways, which can convert hydrogen and carbon dioxide into methanogens; this process is essential for maintaining the rumen’s anaerobic environment. Methanogen colonisation begins at an early stage in lambs, potentially occurring within 2–4 days and reaching an adult stage within 10–14 days [25]; these microorganisms maintain a critical role in early rumen development and exhibit potential as a target for methane mitigation strategies. In the rumen fluid of goats of different ages, Thaumarchaeota, (15%) and Euryarchaeota (82%) were the predominant phyla, although archaeal communities showed less variation with age.

Fungi colonisation follows a three-phase model (an initial phase, a transition phase, and a relatively stable phase) [26], showing significant heterogeneity in early life, while gastrointestinal fungal communities become more homogeneous as lambs progress towards maturity. Some genera were identified both in the rumen and the rectum; namely, *Acremonium*, *Microascus*, *Valsonectria*, *Myrmecridium*, *Scopulariopsis*, *Myrothecium*, *Saccharomyces*, and *Stephanonectria*.

The proportion of protozoa ranges from 20 to 50%, with ciliates and flagellates representing the most common group in the rumen microbial community [27]. In particular, flagellates are present in newborn lambs; however, their abundance gradually decreases with age, while ciliates dominate the protozoa group after maturation [28].

## 4. Extrinsic Factors That Influence Early Gastrointestinal Tract Colonisation

The colonisation of the rumen, and more generally, of the gastrointestinal tract (GIT), is influenced by several factors, both intrinsic (for example age) and extrinsic, primarily dietary. Accordingly, the following sections focus on the role of selected extrinsic factors (dietary patterns and potential supplements) [29].

While the focus of this review is on the rumen microbiota, it is not always possible to distinguish between factors affecting only the rumen microbiota and those exerting significant effects on other segments of the gut; moreover, most studies address a comprehensive modulation of the gut microbiota. Therefore, in the following sections, details concerning the rumen microbiota as well as the overall composition of the gut microbiota are discussed.

### 4.1. Maternal Sources and Diet

The initial neonatal microbiome colonisation largely results from interactions between maternal and offspring microbial populations, which may occur through exposure to the mother’s vagina, udder skin, and breast milk [30]. Limited data are available for lambs, and some information may be inferred by analysing the composition of gut and rumen microbiota in polygastric animals more broadly.

It is generally accepted that the use of milk, milk-replacer, or grains may modulate the rumen microbiota differently; for example, in cows Park et al. [31] reported that the predominant bacteria in the rumen content of animals that received no milk replacer shifted from *Prevotella* to *Bacteroides* six weeks after birth.

Following weaning, diet is considered the primary driver of ruminal microbial assembly, affecting ruminal fermentation patterns, and overall digestive efficiency; indeed, solid feeds, containing complex carbohydrates, proteins, lipids, and secondary metabolites, contribute to the creation of ecological niches for different microbial guilds [32]. For example, diets rich in starch and rapidly fermentable carbohydrates promote the growth of amylolytic and saccharolytic taxa such as *Prevotella*, *Succiniclasticum*, and members of the Veillonellaceae [33], at least in cows; however, these findings may be used to extrapolate potential rumen and gut microbiota composition in lambs. *Prevotella*, *Succiniclasticum*, and members of the Veillonellaceae family are responsible for the production of total volatile fatty acids (VFAs), with increased propionate and butyrate proportions and a reduced acetate-to-propionate ratio; these processes generally improve energetic efficiency and promote tissue accretion [34,35].

Conversely, when the proportion of concentrate is excessively high, the rapid fermentation of starch reduces ruminal pH and promotes lactate-producing bacteria (e.g., *Streptococcus bovis* or *Lactobacillus* spp.), which may be responsible for a syndrome known as subacute ruminal acidosis (SARA). As a consequence, SARA inhibits fibrolytic bacteria, like *Fibrobacter succinogenes* and *R. flavefaciens*, thereby affecting the balance of microbial networks [36]. A diet rich in fibre promotes fibrolytic bacterial groups, stabilises ruminal pH, and contributes to a balanced microbial ecosystem [37].

In lambs, proteins plays a major role, and may selectively modulate specific taxa, like *Christensenellaceae*_R-7_group and *Ruminococcus* [38]. Moreover, feed form and structure may represent significant drivers of microbial ecology, as reported by Li et al. [39] who found that lambs receiving pelleted total mixed rations (PTMRs) had a lower alpha diversity but an increased abundance of fibrolytic bacteria, contributing to improved fibre degradation and growth.

In polygastric animals, some dietary additives may also modulate the rumen microbiota. Live yeast (*Saccharomyces cerevisiae*) has been widely reported to enhance the abundance of fibrolytic bacteria and mitigate pH drops by scavenging oxygen and stimulating lactate-utilising microbes [40].

Plant secondary metabolites (condensed tannins and saponins) and essential oils may modulate ruminal fermentation and selectively inhibit specific microbial taxa, like methane-producing archaea and protozoa [41].

Data collected through metagenomic and metabolomic studies suggest that dietary modulation during the post-weaning period has long-lasting effects on microbial functional pathways, including carbohydrate-active enzymes, short-chain fatty acid production, and biohydrogenation processes [42]. Therefore, the post-weaning period represents a strategic window for guiding the maturation of the ruminal microbiota in lambs. By balancing energy density, providing adequate effective fibre, optimising protein supply, and judiciously incorporating functional additives, it is possible to cultivate a stable and efficient ruminal ecosystem, which in turn may contribute to improved animal health, reduced greenhouse gas emissions, and more sustainable production systems [43].

To provide a concise overview of the key ideas discussed in this section, Table 2 summarises the main dietary drivers of ruminal microbiota modulation in lambs, highlighting the associated microbial responses and their functional implications.

### 4.2. Prebiotics and Probiotics

To stabilise a healthy gut microbiome and increase sheep growth rates, feed additives are used to support beneficial microbes (prebiotics and probiotics, or a combination thereof) [44]. The rationale behind the use prebiotics in animal feeding is that the bacteria known to enhance gut function (lactic acid bacteria, bifidobacteria, and others) preferentially digest non-digestible dietary components such as oligosaccharides [45]; these bacteria are responsible for direct health effects, increasing host microorganism balance. The general mode of action of prebiotics in the GIT includes the enhancement of absorption, protein and fibre digestion, and energy metabolism through the alteration of gut microbiota, as well as reduced mortality associated with pathogenic microorganisms.

Although the classical definition of prebiotics includes fructans with various degrees of polymerisation, mannans, and galactans [46], the updated definition proposed by the Consensus Panel of The International Scientific Association for Probiotics and Prebiotics (ISAPP) [47] suggests that other compounds may also be considered as prebiotics (phenols and other phytochemicals, conjugated linolenic acid, and polyunsaturated fatty acids), as fermentation is no longer considered the only mechanism through which these compounds may exert a beneficial effect in the host gut [48].

An example of a prebiotic formulation for growing lambs is provided in the study by Quijada et al. [49], who evaluated milk supplementation with a combination of fructooligosaccharides (FOSs) derived from sugar beet and garlic residues and observed a weight gain and a modulation of the faecal microbiota, including higher abundances of *Bifidobacterium*, *Enterococcus*, *Lactobacillus*, and *Veillonella*.

In another study, Chashnide et al. [50] used a combination of MOS (Mannan-oligosaccharide) and BG (β-glucans), extracted from *Saccharomyces cerevisiae*, together with a peptide preparation from soybean, to feed 72 newborn lambs; although gut microbiota composition was not assessed, related indicators, such as the immune response, were evaluated, revealing a significant improvement in immune function.

A second approach involves probiotic supplementation, as several researchers have reported a wide range of actions of probiotic microorganisms, involving direct effects on rumen pH and protozoa, overall digestion, greenhouse gas emissions, small intestine flow rate, and growth performance [51]. Regarding growth performance, Antunović et al. [52] and Whitley et al. [53], for example, reported the positive effects of probiotic supplementation on nutrient intake, body weight gain, and feed conversion rate (FCR), which may be linked to improved cellulolytic activity, enhanced fibre degradation [54], and increased microbial protein synthesis leading to greater post-ruminal amino acid digestibility [55,56], or the ability of probiotics to adhere to the intestinal mucosa and prevent pathogen adhesion [57]. Many authors have suggested that the underlying mechanism for most positive effects of probiotics in lambs is the modulation of the rumen microbiota and, more generally, the gut microbiota. For example, inoculation with supernatant from ruminal solids (SRS) with a microbiota under eubiotic conditions increased gut bacterial richness and community, downregulated the *Firmicutes*/*Bacteroidetes* ratio, and increased the abundance of beneficial microorganisms (*Bacteroidetes*, *Spirochaetota*, and *Fibrobacterota*), while reducing the abundance of *Fusobacteriota*, compared with the control group [58].

A positive modulation of the gut microbiota was also reported by Dou et al. [59], who evaluated the effects of *Cl. butyricum* on skeletal muscle development, gastrointestinal microbiota, and meat quality in lambs. The authors hypothesised that the positive effects on meat quality were linked to significant modulation of specific genera; metagenomic analyses revealed higher abundaces of *Petrimonas* spp. and *P. brevis* in the rumen, and *Lachnoclostridium* spp., *Alloprevotella* spp., and *Prevotella* spp. in the faeces, associated with increased butyric acid and valeric acid levels.

A positive modulation of lamb gut microbiota may also be associated with spore-forming bacteria, as reported by Devyatkin et al. [60], who used probiotic strains of *Bacillus subtilis* and *B. licheniformis*. The researchers observed a significant in *Lactobacillus* and *Bifidobacterium* levels and a decrease in *E. coli*, and *Enterococcus*, along with improved weight gain, increased serum protein levels, and reduced bilirubin and cholesterol.

### 4.3. Synbiotics

Most studies do not use prebiotics or probiotics individually, but synbiotics. Synbiotics are mixtures of probiotics and prebiotics that are beneficial to the host by improving the survival and implantation of live microbial dietary supplements in the GIT, stimulating the growth and metabolism of one or a limited number of health-promoting bacteria, and thus improving the welfare of the host. The term synbiotic refers to the synergistic combination of probiotics and prebiotics, as improving the survival of probiotic bacteria in the gastrointestinal tract is considered the primary purpose of this type of combination [61].

MOS is a commercial prebiotic product, generally found in the cell wall of *S. cerevisiae*; when combined with BG, MOS has been reported to improve average daily gain, feeding efficiency, and nutrient absorption. In addition, these values were slightly higher than those observed in probiotic-supplemented feed [62].

According to the study by El-Mehanna et al. [63], the average daily growth and final weight of the growing lambs fed with prebiotics or probiotics were higher better than those of the control groups. In the review by Svitáková et al. [64], the effects of diets supplemented with a probiotic strain of *S. cerevisiae*, and prebiotics MOS + BG in lambs were reported, with a postulated increase in the digestibility of most all nutrients following prebiotic and probiotic supplementation.

In sheep, the albumin (A) fraction increased by 9.7–14.5% in group 2, along with an increase in the albumin–globin (A/G) ratio. As a result of the changes in the Globulin G fraction, which functions as an antibody carrier and provides protection against the attack of infectious agents, growth showed a 10.8% increase [65].

Fouhse et al. [66] demonstrated a synergistic effect of the association between *Lactobacillus paracasei* and fructooligosaccharides (FOS) on the gut microbiota of goat calves. Their findings indicated that the synbiotic group showed modest increase in overall bacterial counts, and the abundance of various anaerobic and aerobic bacterial genera, including *Lactobacillus* and *Bifidobacterium*, a response typical of probiotic supplementation. In addition, they observed a significant reduction in pathogenic bacteria, such as *Escherichia coli*, Enterobacteriaceae, and *Clostridium*, emphasising the potential of synbiotics to improve gut health conditions and microbial balance in livestock. Similarly, Fischer et al. [67] evaluated the efficacy of a commercial synbiotic product (CalfPro™) in calves. Their research demonstrated that the symbiotic exhibited growth-stimulating potential comparable to adriamycin, an antibiotic growth promoter. Finally, Qiu et al. [17] demonstrated the effects of a synbiotic containing *Lactobacillus* spp., with the addition of lactose.

Table 3 presents a tentative compilation of synbiotic, prebiotic, and probiotic preparations, illustrating some of the most promising evidence of direct or indirect manipulation of the gut microbiota in lambs and neonatal animals. Accordingly, Table 4 summarises the main effects of these supplements on the composition and activity of the intestinal microbiota of lambs and neonatal animals.

## 5. Conclusions and Future Perspectives

Diet is a key driver for the modulation and maturation of the ruminal microbiota in lambs; factors such as the earliest phases of life, colostrum/milk exposure, and the timely introduction of solid feeds are crucial and exert long-lasting effects on microbial ecology, immune development, fermentation pathways, and nutrient utilisation. Other factors to consider include the balance between concentrates and effective fibre, the level and ruminal degradability of protein, the physical form of the diet, and the use of functional additives. An approach that has gained considerable attention in recent years is the use of prebiotics, probiotics, and synbiotics to improve microbial stability, increase fibre-degrading capacity, and enhance overall animal performance. However, complex interactions between microbial strains, dietary composition, and host physiology, may lead to variable outcomes, and there remains a need for standardised methodologies and well-replicated experimental designs. Looking ahead, multiomics technologies (metagenomics, metatranscriptomics, and metabolomics), coupled with detailed rumen phenotyping, may contribute to elucidating the relationships among diet, microbiota establishment, and host responses. In turn, these tools may also allow for the identification of critical time frames of microbial ‘imprinting’ during early life and the determination of longer-term effects of these imposed microbial imprints under varying production conditions.

In conclusion, understanding these interactions at a more mechanistic level will be crucial for advancing sustainable grazing management and improving overall ruminant production system efficiency. Future research integrating multiomics approaches and longitudinal study designs will be essential to define early-life microbial imprinting mechanisms and to develop nutritional strategies for optimising ruminant performance and welfare.

## Figures and Tables

**Table 1:** Rumen colonisation from birth to weaning. The table is an original contribution by the authors, produced relying on different references.

	Age (Days)				
	2	7	14	28	42
**Bacteria**					
Proteobacteria	>50%	10–30%	10–30%	10–30%	10–30%
Bacteroidetes	10–30%	>50%	>50%	>50%	>50%
Firmicutes	10–30%	10–30%	10–30%	10–30%	10–30%
Actinobacteria	2–10%	2–10%	2–10%	2–10%	2–10%
Fusobacteria	2–10%	2–10%	<2%	<2%	<2%
Spirochaetes	<2%	<2%	<2%	<2%	<2%
Fibrobacteres	<2%	<2%	<2%	<2%	<2%
Tenericutes	-	<2%	<2%	-	-
Elusimicrobia	-	-	<2%	<2%	2–10%
Lentisphaerae	-	-	<2%	<2%	<2%
**Archaea**					
Euryarchaeota	<2%	<2%	2–10%		
**Fungi**					
*Aspergillus*				2–10%	10–30%
**Protozoa**	10–30%	10–30%	2–10%	-	-
*Endomorphs*	10–30%	10–30%	2–10%	-	-

**Table 2:** Factors affecting rumen microbiota in weaning and post-weaning periods.

**Early Colonisation and Pre-Weaning**	
Sources	Dam’s vagina, udder skin, and colostrum/milk “seeds” microbiota
Breastfeeding	Contributes to immune development and to the establishment of an early microbial ecosystem
Early solid feed introduction	Could limit growth loss during transition phase, and affects microbial assembly
Milk vs. replace	Could determine a shift from *Prevotella* to *Bacteroides*
**Post-Weaning**	
High concentrate/diets rich in starch	Enhancement of amylolytic and saccharolytic taxa Increase in VFA, changes in the ratio acetate vs. propionate, with an increase energy efficiency
Excessive concentrate	Reduction in pH in the rumen, favouring lactate producers and decreasing fibrolytic taxa
Diets rich in fibre	Positive effect on fibrolytic bacteria, pH homeostasis, and microbial diversity
Physical form of diet	Pelleted rations positively modulate fibrolytic bacteria, but could impact on alpha diversity
Additives	Yeasts stabilise pH and have a positive impact on fibrolytic bacteria and on lactate producers Microalgae improve feed efficiency Plant metabolites can impact on methanogenic bacteria and protozoa

**Table 3:** List of probiotics, prebiotics, and synbiotics used for sheep. MOS, mannan oligosaccharides; XOS, xylooligosaccharides; FOS, fructooligosaccharides; GOS, galactooligosaccharides; BG, β-glucans.

Probiotic	Prebiotic	Synbiotic
*Lactobacillus*, *Bifidobacterium*, and their combinations [68,69,70]	Inulin [71]	Lactobacilli + inulin [72]
*Saccharomyces* [73]	GOSs [74]	Lactobacilli + FOS [66]
	FOSs [49]	*S. cerevisiae* + MOS + BG [73]
*Propionibacteria* [75]	XOS [76]	
Rumen extracts [58]	MOS [77]	
*Clostridium butyricum* [59]	BG [77]	
*Bacillus subtilis* [60]	MOS + BG [50]	
*Bacillus licheniformis* [60]		

**Table 4:** Synopsis of the most important changes in the gut microbiota after prebiotic, probiotic, or synbiotic supplementation in lambs and/or newborn infants. +, increase; −, reduction.

**Prebiotics**	
Biodiversity and richness of microbiota	**+**
Faecal levels of *Veillonella*, *Bifidobacterium*, *Lactobacillus*, and *Enterococcus*	**+**
**Probiotics**	
Biodiversity and richness	**+**
*Petrimonas* and *Prevotella* in the rumen	**+**
*Lachnospirillum*, *Alloprevotella* and *Prevotella* in the faeces	**+**
Bacteroidetes, Spirochaetota, and Fibrobacterota	**+**
*Lactobacillus* and *Bifidobacterium*	**+**
Fusobacteria	**−**
*Escherichia coli*, and *Enterococcus*	**−**
**Synbiotics**	
Biodiversity	**+**
*Lactobacillus* and *Bifidobacterium*	**+**
Enterobacteriaceae	**−**
*Clostridium*	**−**

## Data Availability

All the data generated are available in the manuscript.

## References

[1] Elghandour M.M., Khusro A., Adegbeye M.J., Tan Z., Abu Hafsa S., Greiner R., Ugbogu E., Anele U.Y., Salem A.Z. (2020). Dynamic role of single-celled fungi in ruminal microbial ecology and activities. J. Appl. Microbiol..

[2] Harmon D., Swanson K. (2020). Nutritional regulation of intestinal starch and protein assimilation in ruminants. Animals.

[3] He C., Lei J., Yao Y., Qu X., Chen J., Xie K., Wang X., Yi Q., Xiao B., Guo S. (2021). Black soldier fly (*Hermetia illucens*) larvae meal modulates intestinal morphology and microbiota in Xuefeng black-bone chickens. Front. Microbiol..

[4] Huaiquipán R., Quiñones J., Díaz R., Velásquez C., Sepúlveda G., Velázquez L., Paz E.A., Tapia D., Cancino D., Sepúlveda N. (2023). Effect of experimental diets on the microbiome of productive animals. Microorganisms.

[5] Kong F., Liu Y., Wang S., Zhang Y., Wang W., Yang H., Lu N., Li S. (2022). Nutrient digestibility, microbial fermentation, and response in bacterial composition to methionine dipeptide: An in vitro study. Biology.

[6] Moraïs S., Mizrahi I. (2019). The road not taken: The rumen microbiome, functional groups, and community states. Trends Microbiol..

[7] Chen S., Luo S., Yan C. (2021). Gut microbiota implications for health and welfare in farm animals: A review. Animals.

[8] Lv X., Chai J., Diao Q., Huang W., Zhuang Y., Zhang N. (2019). The signature microbiota drive rumen function shifts in goat kids introduced to solid diet regimes. Microorganisms.

[9] Kou X., Ma Q., Liu Y., Zahoor Khan M., Bu W., Chen W., Liu X., Wang C., Li Y. (2024). Exploring the effect of gastrointestinal *Prevotella* on growth performance traits in livestock animals. Animals.

[10] Krause D.O., Dalrymple B.P., Smith W.J., Mackie R.I., McSweeney C.S. (1999). 16S rDNA sequencing of *Ruminococcus albus* and *Ruminococcus flavefaciens*: Design of a signature probe and its application in adult sheep. Microbiology.

[11] Zhang H., Siao M., Huang H., Wang S., Ma L., Wang H., Hu L., Wei K., Zhu R. (2018). The dynamic distribution of small-tail Han sheep microbiota across different intestinal segments. Front. Microbiol..

[12] Jin S., Zhang Z., Zhang G., He B., Qin Y., Yang B., Yu Z., Wang J. (2023). Maternal rumen bacteriota shapes the offspring rumen bacteriota, affecting the development of young ruminants. Microbiol. Spectr..

[13] Diaz J., Reese A.T. (2021). Possibilities and limits for using the gut microbiome to improve captive animal health. Anim. Microbiome.

[14] Yáñez-Ruiz D.R., Abecia L., Newbold C.J. (2015). Manipulating rumen microbiome and fermentation through interventions during early life: A review. Front. Microbiol..

[15] Jiao J., Li X., Beauchemin K.A., Tan Z., Tang S., Zhou C. (2015). Rumen development process in goats as affected by supplemental feeding v. grazing: Age-related anatomic development, functional achievement and microbial colonisation. Br. J. Nutr..

[16] Bi Y., Tu Y., Zhang N., Wang S., Zhang F., Suen G., Shao D., Li S., Diao Q. (2021). Multiomics analysis reveals the presence of a microbiome in the gut of fetal lambs. Gut.

[17] Qiu Y., Liu S., Hou L., Li K., Wang L., Gao K., Yang X., Jiang Z. (2021). Supplemental choline modulates growth performance and gut inflammation by altering the gut microbiota and lipid metabolism in weaned piglets. J. Nutr..

[18] Malmuthuge N., Liang G., Guan L.L. (2019). Regulation of rumen development in neonatal ruminants through microbial metagenomes and host transcriptomes. Genome Biol..

[19] Yin X., Ji S., Duan C., Tian P., Ju S., Yan H., Zhang Y., Liu Y. (2021). Age-related changes in the ruminal microbiota and their relationship with rumen fermentation in lambs. Front. Microbiol..

[20] Dunière L., Ruiz P., Lebbaoui Y., Guillot L., Bernard M., Forano E., Chaucheyras-Durand F. (2023). Effects of rearing mode on gastro-intestinal microbiota and development, immunocompetence, sanitary status and growth performance of lambs from birth to two months of age. Anim. Microbiome.

[21] Rey M., Enjalbert F., Combes S., Cauquil L., Bouchez O., Monteils V. (2014). Establishment of ruminal bacterial community in dairy calves from birth to weaning is sequential. J. Appl. Microbiol..

[22] Zhang K., Li B., Guo M., Liu G., Yang Y., Wang X., Chen Y., Zhang E. (2019). Maturation of the goat rumen microbiota involves three stages of microbial colonization. Animals.

[23] Wang L., Zhang K., Zhang C., Feng Y., Zhang X., Wang X., Wu G. (2019). Dynamics and stabilization of the rumen microbiome in yearling Tibetan sheep. Sci. Rep..

[24] Zhang Y., Choi S., Nogoy K., Liang S. (2021). The development of the gastrointestinal tract microbiota and intervention in neonatal ruminants. Animals.

[25] Wang L., Xu Q., Kong F., Yang Y., Wu D., Mishra S., Li Y. (2016). Exploring the goat rumen microbiome from seven days to two years. PLoS ONE.

[26] Yin X., Ji S.-K., Duan C.-H., Tian P.-Z., Yan H., Zhang Y.-J., Liu Y.-Q. (2022). Dynamic change of fungal community in the gastrointestinal tract of growing lambs. J. Integr. Agric..

[27] Cholewińska P., Czyż K., Nowakowski P., Wyrostek A. (2020). The microbiome of the digestive system of ruminants—A review. Anim. Health Res. Rev..

[28] Li B., Zhang K., Li C., Wang X., Chen Y., Yang Y. (2019). Characterization and comparison of microbiota in the gastrointestinal tracts of the goat (*Capra hircus*) during preweaning development. Front. Microbiol..

[29] Cholewińska P., Górniak W., Wojnarowski K. (2021). Impact of selected environmental factors on microbiome of the digestive tract of ruminants. BMC Vet. Res..

[30] Ramayo-Caldas Y., Zingaretti L., Popova M., Estellé J., Bernard A., Pons N., Bellot P., Mach N., Rau A., Roume H. (2020). Identification of rumen microbial biomarkers linked to methane emission in Holstein dairy cows. J. Anim. Breed. Genet..

[31] Park S.J., Beak S.-H., Kim S.Y., Jeong I.H., Piao M.Y., Kang H.J., Fassah D.M., Na S.W., Yoo S.P., Baik M. (2018). Genetic, management, and nutritional factors affecting intramuscular fat deposition in beef cattle—A review. Asian-Australas. J. Anim. Sci..

[32] Duncan S.H., Conti E., Ricci L., Walker A.W. (2023). Links between diet, intestinal anaerobes, microbial metabolites and health. Biomedicines.

[33] Zhang R., Liu J., Jiang L., Mao S. (2020). Effect of high-concentrate diets on microbial composition, function, and the VFAs formation process in the rumen of dairy cows. Anim. Feed Sci. Technol..

[34] Van Houtert M. (1993). The production and metabolism of volatile fatty acids by ruminants fed roughages: A review. Anim. Feed Sci. Technol..

[35] Cheng X., Liang Y., Ji K., Feng M., Du X., Jiao D., Wu X., Zhong C., Cong H., Yang G. (2025). Enhanced propionate and butyrate metabolism in cecal microbiota contributes to cold-stress adaptation in sheep. Microbiome.

[36] Sun J., Zhang L., Loh K.-C. (2021). Review and perspectives of enhanced volatile fatty acids production from acidogenic fermentation of lignocellulosic biomass wastes. Bioresour. Bioprocess..

[37] Wang L., Li Y., Zhang Y., Wang L. (2020). The effects of different concentrate-to-forage ratio diets on rumen bacterial microbiota and the structures of Holstein cows during the feeding cycle. Animals.

[38] Cai Y., Zou J., Zhou Y., Yang J., Wang C., Mao H. (2025). Nitrogen utilization and ruminal microbiota of Hu lambs in response to varying dietary metabolizable protein levels. Animals.

[39] Li S., Du M., Zhang C., Wang Y., Lee Y., Zhang G. (2022). Diet type impacts production performance of fattening lambs by manipulating the ruminal microbiota and metabolome. Front. Microbiol..

[40] Chaucheyras-Durand F., Ameilbonne A., Bichat A., Mosoni P., Ossa F., Forano E. (2016). Live yeasts enhance fibre degradation in the cow rumen through an increase in plant substrate colonization by fibrolytic bacteria and fungi. J. Appl. Microbiol..

[41] Ku-Vera J.C., Jiménez-Ocampo R., Valencia-Salazar S.S., Montoya-Flores M.D., Molina-Botero I.C., Arango J., Gómez-Bravo C.A., Aguilar-Pérez C.F., Solorio-Sánchez F.J. (2020). Role of secondary plant metabolites on enteric methane mitigation in ruminants. Front. Vet. Sci..

[42] Gaio D., DeMaere M.Z., Anantanawat K., Chapman T.A., Djordjevic S.P., Darling A.E. (2021). Post-weaning shifts in microbiome composition and metabolism revealed by over 25,000 pig gut metagenome-assembled genomes. Microb. Genom..

[43] Wang S., Chai J., Zhao G., Zhang N., Cui K., Bi Y., Ma T., Tu Y., Diao Q. (2022). The temporal dynamics of rumen microbiota in early weaned lambs. Microorganisms.

[44] Markowiak P., Śliżewska K. (2018). The role of probiotics, prebiotics and synbiotics in animal nutrition. Gut Pathog..

[45] Jha R., Fouhse J.M., Tiwari U.P., Li L., Willing B.P. (2019). Dietary fiber and intestinal health of monogastric animals. Front. Vet. Sci..

[46] Gibson G.R., Roberfroid M.B. (1995). Dietary modulation of the human colonic microbiota: Introducing the concept of prebiotics. J. Nutr..

[47] Gibson G.R., Hutkins R., Sanders M.E., Prescott S.L., Reimer R.A., Salminen S.J., Scott K., Stanton C., Swanson K.S., Cani P.D. (2017). Expert consensus document: The International Scientific Association for Probiotics and Prebiotics (ISAPP) consensus statement on the definition and scope of prebiotics. Nat. Rev. Gastroenterol. Hepatol..

[48] Bevilacqua A., Campaniello D., Speranza B., Racioppo A., Sinigaglia M., Corbo M.R. (2024). An update on prebiotics and on their health effects. Foods.

[49] Quijada N.M., Bodas R., Lorenzo J.M., Schmitz-Esser S., Rodríguez-Lázaro D., Hernández M. (2020). Dietary supplementation with sugar beet fructooligosaccharides and garlic residues promotes growth of beneficial bacteria and increases weight gain in neonatal lambs. Biomolecules.

[50] Chashnidel Y., Bahari M., Yansari A.T., Kazemifard M. (2020). The effects of dietary supplementation of prebiotic and peptide on growth performance and blood parameters in suckling Zell lambs. Small Rum. Res..

[51] El-Trwab A., Youssef I., Bakr H., Fthenakis G., Giadinis N. (2016). Role of probiotics in nutrition and health of small ruminants. Pol. J. Vet. Sci..

[52] Antunović Z., Šperanda M., Amidžić D., Šerić V., Stainer Z., Domačinović M., Boli F. (2006). Probiotic application in lambs nutrition. Krmiva Časopis O Hranidbi Zivotinj. Proizv. I Tehnol. Krme.

[53] Whitley N.C., Cazac D., Rude B., Jackson-O’Brien D., Parveen S. (2009). Use of a commercial probiotic supplement in meat goats. J. Anim. Sci..

[54] Russell J.B., Wilson D.B. (1996). Why are ruminal cellulolytic bacteria unable to digest cellulose at low pH?. J. Dairy Sci..

[55] Erasmus L., Botha P., Kistner A. (1992). Effect of yeast culture supplement on production, rumen fermentation, and duodenal nitrogen flow in dairy cows. J. Dairy Sci..

[56] Chaucheyras-Durand F., Walker N., Bach A. (2008). Effects of active dry yeasts on the rumen microbial ecosystem: Past, present and future. Anim. Feed Sci. Technol..

[57] Seo J.K., Kim S.-W., Kim M.H., Upadhaya S.D., Kam D.K., Ha J.K. (2010). Direct-fed microbials for ruminant animals. Asian-Australas. J. Anim. Sci..

[58] Fu L., Liu L., Zhang L., Hu Y., Zeng Y., Ran Q., Zhou Y., Zhou P., Chen J., Loor J.J. (2024). Inoculation of newborn lambs with ruminal solids derived from adult goats reprograms the development of gut microbiota and serum metabolome and favors growth performance. J. Agric. Food Chem..

[59] Dou L., Liu C., Chen X., Yang Z., Hu G., Zhang M., Sun L., Su L., Zhao L., Jin Y. (2023). Supplemental Clostridium butyricum modulates skeletal muscle development and meat quality by shaping the gut microbiota of lambs. Meat Sci..

[60] Devyatkin V., Mishurov A., Kolodina E. (2021). Probiotic effect of Bacillus subtilis B-2998D, B-3057D, and Bacillus licheniformis B-2999D complex on sheep and lambs. J. Adv. Vet. Anim. Res..

[61] Ma T., Villot C., Renaud D., Skidmore A., Chevaux E., Steele M., Guan L.L. (2020). Linking perturbations to temporal changes in diversity, stability, and compositions of neonatal calf gut microbiota: Prediction of diarrhea. ISME J..

[62] Priyoatmojo D., Maharani Y., Ansori D., Hardani S., Trinugraha A., Handayani T., Sasongko W., Wahyono T. (2021). Effects of harvesting time on tannin biological activity in sambiloto (*Andrographis paniculata*) leaves and in vitro diet digestibility supplemented with sambiloto leaves. J. Anim. Health Prod..

[63] El-Mehanna S., Abdelsalam M., Hashem N., El-Azrak K., Mansour M., Zeitoun M. (2017). Relevance of probiotic, prebiotic and synbiotic supplementations on hemato-biochemical parameters, metabolic hormones, biometric measurements and carcass characteristics of sub-tropical Noemi lambs. Int. J. Anim. Res..

[64] Svitáková A., Schmidová J., Pešek P., Novotná A. (2014). Recent developments in cattle, pig, sheep and horse breeding—A review. Acta Vet. Brno.

[65] Wang B., Luo Y., Su R., Yao D., Hou Y., Liu C., Du R., Jin Y. (2020). Impact of feeding regimens on the composition of gut microbiota and metabolite profiles of plasma and feces from Mongolian sheep. J. Microbiol..

[66] Fouhse J.M., Smiegielski L., Tuplin M., Guan L.L., Willing B.P. (2017). Host immune selection of rumen bacteria through salivary secretory IgA. Front. Microbiol..

[67] Fischer A., Song Y., He Z., Haines D., Guan L., Steele M. (2018). Effect of delaying colostrum feeding on passive transfer and intestinal bacterial colonization in neonatal male Holstein calves. J. Dairy Sci..

[68] Wójcik R., Małaczewska J., Tobolski D., Miciński J., Kaczorek-Łukowska E., Zwierzchowski G. (2024). The effect of orally administered multi-strain probiotic formulation (*Lactobacillus*, *Bifidobacterium*) on the phagocytic activity and oxidative metabolism of peripheral blood granulocytes and monocytes in lambs. Int. J. Mol. Sci..

[69] Mao H., Ji W., Yun Y., Zhang Y., Li Z., Wang C. (2023). Influence of probiotic supplementation on the growth performance, plasma variables, and ruminal bacterial community of growth-retarded lamb. Front. Microbiol..

[70] Daszkiewicz T., Miciński J., Wójcik R., Tobolski D., Zwierzchowski G., Kobzhassarov T., Ząbek K., Charkiewicz K. (2025). The effect of probiotic supplementation in Kamieniec lambs on meat quality. Small Rumin. Res..

[71] Dhidhik Arifin H., Rianto E., Purbowati E., Muktiani A. (2025). The potential of synbiotics supplementation using gembili inulin as a prebiotic in improving fermentability and productivity of lambs fed diet of different fibre content. Adv. Anim. Vet. Sci..

[72] Zhou Z., Xu X., Luo D., Zhou Z., Zhang S., He R., An T., Sun Q. (2023). Effect of dietary supplementation of *Lactiplantibacillus plantarum* N-1 and its synergies with oligomeric isomaltose on the growth performance and meat quality in Hu sheep. Foods.

[73] Atmaja B.A., Agusetyaningsih I., Ali M.I., Adikara Y.a.R., Hidayatulloh R., Herviyanto D., Lestari W.M., Hutabarat A.L.R., Safitri A.R., Ali A.M. (2025). The potential of *Saccharomyces cerevisiae* as a biological control agent against gastrointestinal nematodes in sheep. J. Anim. Health Prod..

[74] Mwenya B., Santoso B., Sar C., Gamo Y., Kobayashi T., Arai I., Takahashi J. (2004). Effects of including β1–4 galacto-oligosaccharides, lactic acid bacteria or yeast culture on methanogenesis as well as energy and nitrogen metabolism in sheep. Anim. Feed Sci. Technol..

[75] Ebeid H.M., Kholif A.M., Farghly M.S., Khattab M.S.A. (2013). Effect of propionibacteria supplementation to sheep diets on rumen fermentation, nutrients, digestibility and blood metabolites. Sci. Int..

[76] Yang J., Jia W., Zhang B., Sun S., Dou X., Wu Q., Wang Y., Li Y., Ma W., Ren G. (2025). Effects of diet xylooligosaccharide supplementation on growth performance, carcass characteristics, and meat quality of Hu lambs. Foods.

[77] Zapata O., Cervantes A., Barreras A., Monge-Navarro F., González-Vizcarra V.M., Estrada-Angulo A., Urías-Estrada J.D., Corona L., Zinn R.A., Martínez-Alvarez L.G. (2021). Effects of single or combined supplementation of probiotics and prebiotics on ruminal fermentation, ruminal bacteria and total tract digestion in lambs. Small Rumin. Res..

